# Congenital Intestinal Malrotation With Coexistent Jejunal Diverticula in a Septuagenarian Male: A Case Report

**DOI:** 10.7759/cureus.86981

**Published:** 2025-06-29

**Authors:** Muhammad Umer Farooq Siddiqui, Mahnoor Saeed, Muhammad Naeem Khattak, Aamir Khan

**Affiliations:** 1 General Surgery, Khyber Teaching Hospital, Khyber Medical College, Peshawar, PAK

**Keywords:** intestinal malrotation, jejunal diverticula, ladd’s procedure, volvulus, whirlpool sign

## Abstract

Intestinal malrotation is a congenital anomaly with mostly reported cases in neonates, although, in adults, limited cases have been reported in the literature. However, in adults, prompt recognition is of utmost importance to prevent drastic complications like bowel ischemia and death. We present a rare case of coexistence of congenital intestinal malrotation and jejunal diverticula presenting as a volvulus. We present a case of a 78-year-old male with no comorbidities presenting to the emergency department with an acute abdomen, diagnosed as a case of intestinal obstruction in a virgin abdomen, and treated conservatively. Meanwhile, CT abdomen and pelvis showed volvulus of the small intestine, so the patient was taken for exploratory laparotomy, which revealed the small bowel rotation with a high cecum and a narrow mesentery. Ladd’s procedure was performed without any perioperative complications. Few reports of congenital intestinal malrotation in the elderly are presented in the literature. This highlights the importance of evaluating all patients for malrotation if presenting in elderly patients with a virgin acute abdomen to prevent complications like bowel ischemia. Elderly patients presenting with acute intestinal obstruction, if not improving conservatively, should make the clinician think about congenital intestinal malrotation as a differential diagnosis as well, among all other common causes.

## Introduction

Congenital intestinal malrotation is an anomaly which is more common in neonates, although, in adults, it has an incidence of 0.2%-0.5%, most commonly diagnosed radiologically or perioperatively [1]. In adults, it presents either with vague symptoms or remains asymptomatic throughout life until some coexisting diseases or a change in physical condition could trigger a life-threatening complication in the form of volvulus [2]. Another rare entity that can cause symptoms of intestinal obstruction in adults is the presence of jejunal diverticula. Midgut volvulus and malrotation should be considered as a differential diagnosis in a patient presenting with recurrent symptoms of small bowel obstruction [3]. Here, we are discussing the diagnosis and operative management of a 78-year-old male presenting as midgut volvulus due to congenital malrotation of intestine with coexistence of jejunal diverticula.

## Case presentation

A 78-year-old male patient with no known comorbidities presented to the emergency department with the chief complaints of abdominal pain and constipation for five days and vomiting for one day. Abdominal pain was diffuse, severe, and progressively increased over the course of five days, accompanied by a few episodes of vomiting, after which he was brought to the emergency department. The patient reported that his last bowel movements occurred four days ago with passage of flatus and minimal hard stools without any melena, hematochezia, or fever.

According to the patient, he had experienced intermittent abdominal pain, decreased bowel movements, and hard stools on defecation for the last 30 years. He had multiple visits to the nearby general practitioners, where he was advised to use laxatives, tablet Flagyl (metronidazole), and over-the-counter pain medications. He had been using laxatives (syrup Lactulose) and tablet Flagyl (metronidazole) regularly. His body mass index (BMI) was 18.2 kg/m^2^, and he ate small meals. During the day, his diet included fibers (mostly from vegetables and fruits), low protein, and carbohydrates, and he used to skip dinner to avoid abdominal pain at late night. Past medical history included chronic abdominal pain, dengue fever, and smallpox during childhood. No past surgical history was reported. The patient worked as a farmer.

**Figure 1:**
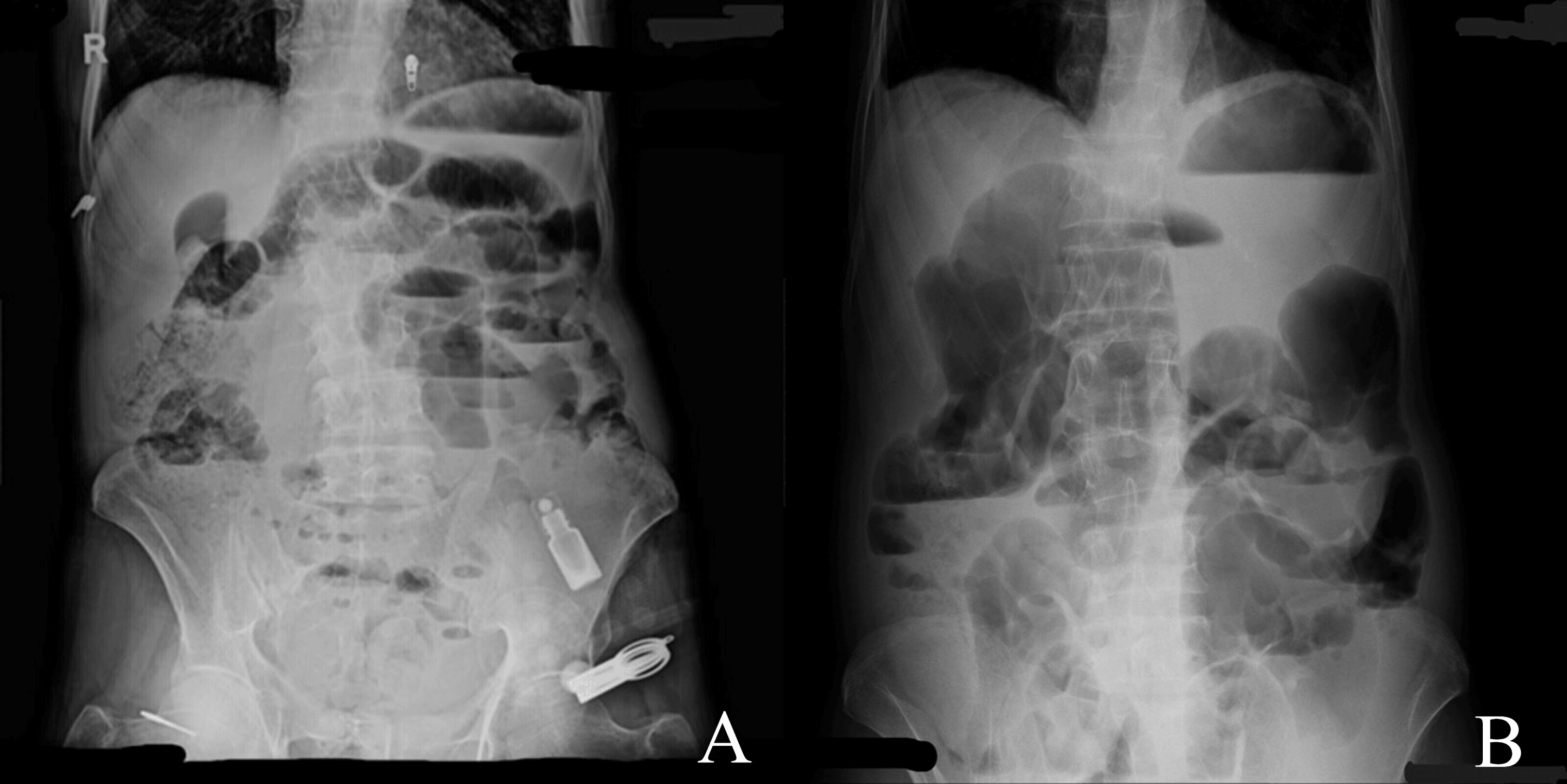
X-ray of erect abdomen. (A) Multiple air fluid levels with dilated bowel loops. (B) Distended bowel loops with fewer air fluid levels after 24 hours of initial resuscitation.

On physical examination in emergency department, the patient was tachycardic, dehydrated but oriented. His abdomen was distended and tender on palpation in all quadrants with rebound tenderness and guarding. Bowel sounds were absent on auscultation. Signs and symptoms were suggestive of acute intestinal obstruction. On digital rectal examination, there were hard impacted stools which were manually evacuated. The patient was admitted and resuscitated with intravenous (IV) fluids, nasogastric tube for decompression, catheterization, antibiotics, IV painkillers, IV proton pump inhibitors (PPIs), and was made nil per os (NPO). All baseline investigations were done along with X-ray erect abdomen (Figures 1A, 1B) and ultrasound abdomen and pelvis and computed tomography (CT) scan of abdomen and pelvis with intravenous contrast. Laboratory tests were unremarkable. Ultrasound report showed moderately dilated bowel loops with to-and-fro fluid movement, a few non-obstructive calculi in the right kidney, a simple cyst in the left kidney measuring 33 x 32 mm, and prostatomegaly (82 g).

**Figure 2:**
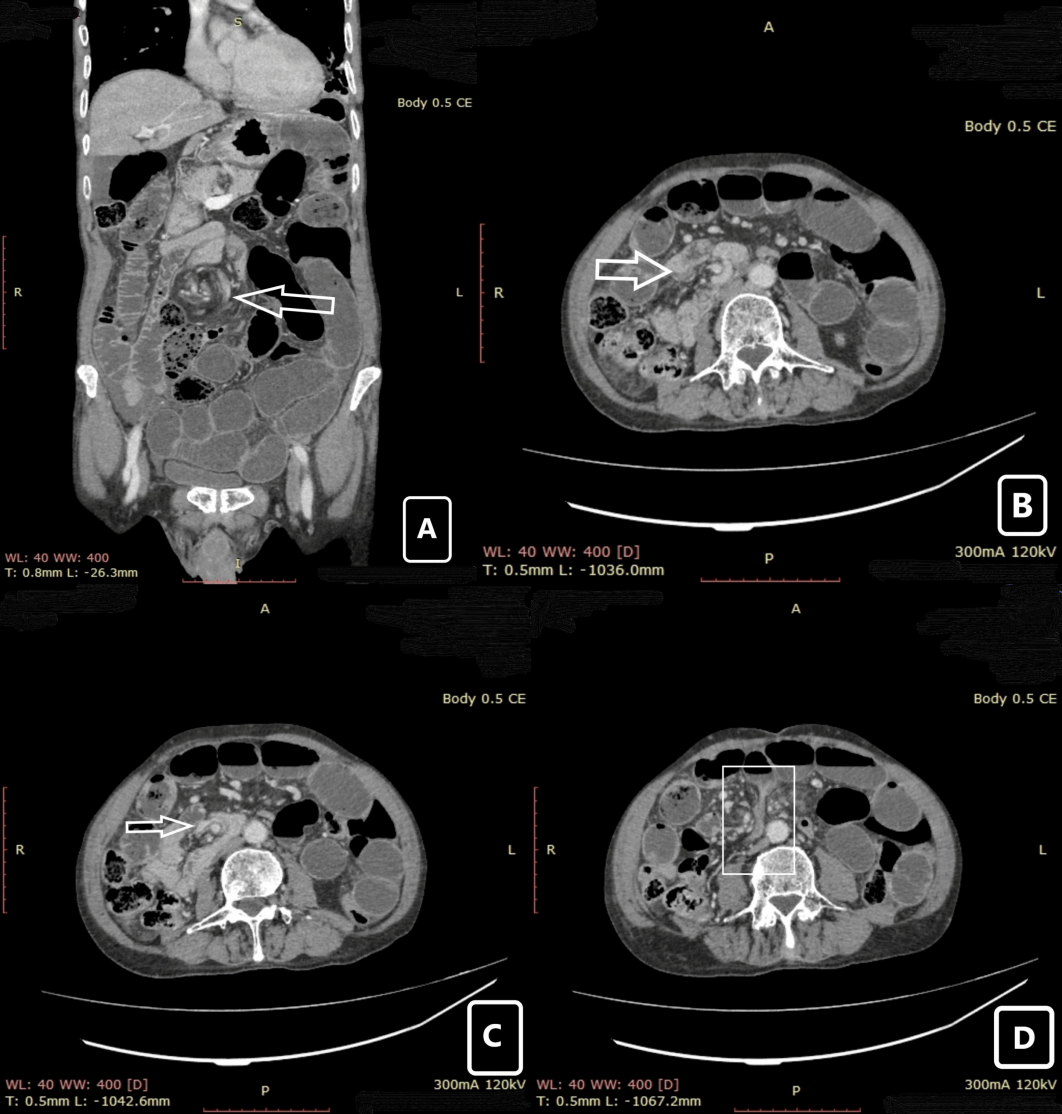
CT abdomen and pelvis (coronal and axial view) images. (A) Whirl pool sign (arrow) in coronal section. (B) Rotatory movement of superior mesenteric vessels (arrow) in axial view. (C) Complete rotation of superior mesenteric vessels (arrow) in axial view. (D) Duodenal beak sign (box) indicating rotation of distal bowel. CE: contrast enhancement, R: right, L: left, WL: window level, WW: window width, T: transverse, A: anterior, P: posterior.

Multidetector CT scan of abdomen and pelvis with intravenous contrast (Figures 2A-2D) revealed small bowel obstruction, signified by dilated and fluid-filled small bowel loops, multiple air-fluid levels, and a transition zone in the right lower lumbar region, where mesenteric twisting around the superior mesenteric vessels was observed, giving the ‘whirlpool’ sign, suggestive of small bowel volvulus. Duodenum and duodenojejunal junction were displaced to the right of midline. Minimal free intraperitoneal fluid was seen with no obvious signs of bowel ischemia.

With the initial resuscitation, the patient’s symptoms slightly improved, but abdomen was moderately tender even after 24 hours of presentation. After discussing the risk and benefits of exploratory laparotomy (with possible bowel resection) with the patient, high-risk consent was taken, and decision was made to proceed with surgical intervention. The patient was taken to operating room (OR), and general anesthesia was administered. Under aseptic conditions, the patient was prepped and draped; a standard midline incision was made; and peritoneum was opened to gain access to the peritoneal cavity. The small intestine was investigated from 'Ligament of Treitz' to hepatic flexure of ascending colon. The complete section of the examined gut was found to be viable with evident peristalsis. Two Ladd's band were identified and divided. Cecum was identified high up in the right hypochondrium. Malrotation of small bowel mesentery with a narrow base was identified which exposed the superior mesenteric vessels. Five jejunal diverticula were also identified. Two-point fixation of the small intestine was performed, with anchoring of the cecum in the right iliac fossa and the duodenojejunal junction in the left upper quadrant. Mesenteric broadening and expansion were performed to reduce the risk of volvulus recurrence. Hemostasis was secured, and layers were closed in reverse order. Antiseptic dressing applied over the wound. The patient was taken to anesthesia recovery room, and after successful recovery, he was shifted back to surgical ward. Post-operatively, the patient was monitored in ward and no complications were noted during his stay in hospital. The patient was allowed oral diet after six hours post-operatively and was discharged after three days. On follow-up visit after seven days, the patient did not complain of any symptoms and had a smooth recovery.

## Discussion

Intestinal malrotation is a congenital anomaly with abnormal positioning of the intestine from the normal 270-degree rotation around the axis of the superior mesenteric artery during the development of the embryo [4]. Malrotation of the intestine can be classified into these three types depending upon the period of interruption along the embryonal development: type 1 (nonrotation), type 2 (only small bowel malrotation), and type 3 (malrotation of duodenum and cecum) [5]. Our patient is included in type 3 because the duodenum-jejunal flexure was located on the right side, compared to the normal midline position and cecum in the upper right quadrant, leading to a narrow base mesentery. The rest of the small and large bowels were in their normal anatomical position.

Congenital malrotation diagnosed in elderly adults is an uncommon presentation [6]. In the non-infant population, it presents itself in the form of chronic colicky abdominal pain, weight loss, bilious or nonbilious emesis, and food intolerance [6]. In our case, the patient was fine from childhood until his late 60s when he started to develop infrequent symptoms of abdominal pain and food intolerance, leading to repeated visits to the emergency department for abdominal pain until presenting with non-resolving abdominal pain which was diagnosed as subacute intestinal obstruction.

The gold standard for diagnosis of intestinal malrotation is the upper gastrointestinal series which is preferred in the pediatrics population [6]. At the same time, oral or intravenous contrast-enhanced CT abdomen and pelvis are the preferred imaging modality in adults [6]. Salient features observed in CT are the ‘whirlpool sign’ seen in intestinal volvulus and the superior mesenteric vein (SMV) rotation sign (where the SMV lies anterior to or right of the superior mesenteric artery) [6]. We performed a multidimensional CT scan of abdomen and pelvis with IV contrast for the patient which showed a whirlpool sign and duodenojejunal flexure to the right side of the midline which favors volvulus of small bowel leading to intestinal obstruction although small and large bowel being in their normal anatomical positions.

The procedure of choice for correcting congenital intestinal malrotation is Ladd’s procedure be it an elective or emergency procedure [7]. This procedure consists of the following steps: delivery of the small bowel and untwisting it counterclockwise, separation of Ladd bands, placing cecum in the right paravertebral gutter, widening of small bowel mesentery, and appendectomy with or without resection depending upon the condition of the bowel [7]. In our case, all steps were followed along with anchoring of the cecum in the right iliac fossa and duodenal jejunal junction in the left upper quadrant to prevent future twisting of mesentery.

Another unique observation in our case was the presence of multiple jejunal diverticula. Jejunal diverticula are a rare type of small bowel diverticula with a prevalence ranging from 0.002% to 5% [8]. Usually, these diverticula are asymptomatic until in rare cases it can cause intestinal obstruction due to the fluid-filled portion and greater weight bearing of the involved segment as compared to the non-involved segment [8]. The operative intervention is a de-rotation procedure in case of a viable small bowel needing no further fixation [8].

## Conclusions

This case illustrates the rare but significant occurrence of congenital intestinal malrotation with jejunal diverticula in an elderly patient. Although typically diagnosed in infancy, it should not be ruled out in adults presenting with symptoms of acute abdominal obstruction which is not relieved with conservative management and should be considered in the differential diagnosis of such patients. Early recognition through imaging and prompt surgical management are critical to prevent any serious complications.
